# THE NIH STAGE MODEL AND STUDY EXAMPLES OF EARLY STAGES IN DEMENTIA CARE INTERVENTION DEVELOPMENT RESEARCH

**DOI:** 10.1093/geroni/igad104.1121

**Published:** 2023-12-21

**Authors:** Debra Dobbs, Hongdao Meng

## Abstract

The NIH’s Stage Model provides a common language to facilitate discussion of intervention development research. According to NIH Stage Model (2022), “an examination of the mechanisms of behavior change or the principles underlying an intervention is encouraged by the NIH Stage Model in every Stage of behavioral intervention development.” The five papers in this symposium focus on early-stage intervention development by refinement, adaptation and pilot testing (Stage 1) related to dementia and palliative care (PC) clinical trials. The first paper is a cluster trial of a PC education program for nurses (N=23) in 10 assisted living communities that tested the mechanism of staff self-efficacy in increases in advance care planning discussions with family members of residents living with dementia. The second paper is a clinical trial that tested the feasibility of Companion Robots in live-alone Korean-American older adults (N=30) to determine which features help improve health behaviors (e.g. medication adherence, daily exercises). The third paper is a single-site clinical trial pilot study developed with stakeholders which tested the feasibility of a palliative care community health worker support program for hospitalized minoritized persons living with dementia and their caregivers. The fourth paper adapts an evidence-based e-PainSupport intervention for use with dementia caregivers to report patient pain and administration of analgesics. The fifth paper is a protocol to test the feasibility and acceptability of a culturally sensitive caregiver peer support program (Pair2Care) in current (n=15) and trained former (n=15) African-American dementia caregivers. The discussant will highlight ways to improve clinical trial mechanisms.

